# TGFβ-induced switch from adipogenic to osteogenic differentiation of human mesenchymal stem cells: identification of drug targets for prevention of fat cell differentiation

**DOI:** 10.1186/s13287-016-0375-3

**Published:** 2016-08-26

**Authors:** Everardus J. van Zoelen, Isabel Duarte, José M. Hendriks, Sebastian P. van der Woning

**Affiliations:** 1 Department of Cell and Applied Biology, Faculty of Science, Radboud University Nijmegen, PO Box 9010, 6500 GL Nijmegen, The Netherlands; 2 Present Address: Department of Cell and Applied Biology, Radboud University Nijmegen, Heyendaalseweg 135, 6525 AJ Nijmegen, The Netherlands; 3 Present Address: Systems Biology and Bioinformatics Laboratory (SysBioLab), University of Algarve, Faro, Portugal; 4 Present Address: Department of Physical Organic Chemistry, Radboud University Nijmegen, Nijmegen, The Netherlands; 5 Present Address: ARGENX BVBA, Technologiepark 30, B-9052 Zwijnaarde, Belgium

**Keywords:** Mesenchymal stem cell, Adipogenesis, Osteogenesis, Osteoporosis, Bone marrow, Histone deacetylase, Metalloproteinase, Aldo-keto reductase

## Abstract

**Background:**

Patients suffering from osteoporosis show an increased number of adipocytes in their bone marrow, concomitant with a reduction in the pool of human mesenchymal stem cells (hMSCs) that are able to differentiate into osteoblasts, thus leading to suppressed osteogenesis.

**Methods:**

In order to be able to interfere with this process, we have investigated in-vitro culture conditions whereby adipogenic differentiation of hMSCs is impaired and osteogenic differentiation is promoted. By means of gene expression microarray analysis, we have investigated genes which are potential targets for prevention of fat cell differentiation.

**Results:**

Our data show that BMP2 promotes both adipogenic and osteogenic differentiation of hMSCs, while transforming growth factor beta (TGFβ) inhibits differentiation into both lineages. However, when cells are cultured under adipogenic differentiation conditions, which contain cAMP-enhancing agents such as IBMX of PGE2, TGFβ promotes osteogenic differentiation, while at the same time inhibiting adipogenic differentiation. Gene expression and immunoblot analysis indicated that IBMX-induced suppression of HDAC5 levels plays an important role in the inhibitory effect of TGFβ on osteogenic differentiation. By means of gene expression microarray analysis, we have investigated genes which are downregulated by TGFβ under adipogenic differentiation conditions and may therefore be potential targets for prevention of fat cell differentiation. We thus identified nine genes for which FDA-approved drugs are available. Our results show that drugs directed against the nuclear hormone receptor PPARG, the metalloproteinase ADAMTS5, and the aldo-keto reductase AKR1B10 inhibit adipogenic differentiation in a dose-dependent manner, although in contrast to TGFβ they do not appear to promote osteogenic differentiation.

**Conclusions:**

The approach chosen in this study has resulted in the identification of new targets for inhibition of fat cell differentiation, which may not only be relevant for prevention of osteoporosis, but also of obesity.

## Background

Human mesenchymal stem cells (hMSCs) from bone marrow have the ability to differentiate into cells from multiple lineages, including osteoblasts and adipocytes. The commitment of hMSCs towards either the osteogenic or adipogenic lineage depends on the local availability of growth factors and hormones, which are able to activate lineage-specific transcriptional regulators [1]. Patients suffering from osteoporosis show an increased number of adipocytes in their bone marrow, concomitant with a reduction in the pool of hMSCs that are able to differentiate into osteoblasts, thus leading to suppressed osteogenesis [2, 3]. It is still unclear to what extent this age-related increase in differentiation of hMSCs towards adipocytes results from intrinsic changes in the stem cells or from alterations in the microenvironment of the bone marrow [1, 4]. These observations have recently stirred increasing interest in anabolic therapies for osteoporosis, whereby osteogenic differentiation of hMSCs is stimulated by preventing adipogenic differentiation [5, 6].

Most information about the signaling pathways that are required for osteogenic and adipogenic differentiation of hMSCs has come from in-vitro studies, whereby cells are treated with specific combinations of growth factors and hormones [7]. Differentiation into both lineages requires treatment of monolayer cells with dexamethasone (DEX) and is enhanced by the presence of bone morphogenetic proteins (BMPs). Osteogenic differentiation is obtained by the additional presence of ascorbate and β-glycerophosphate, whereas adipogenic differentiation requires treatment with insulin, 3-isobutyl-1-methylxanthine (IBMX), and the PPARG activator rosiglitazone. Our previous data have indicated that under these experimental conditions at least 75 % of the hMSCs differentiate into either osteoblasts or adipocytes [7].

Activation of the RUNX2 nuclear transcription factor appears to be essential for the osteogenic pathway of hMSCs, while the adipogenic pathway requires the transcriptional activity of the PPARG nuclear hormone receptor [1]. Evidence has been presented that transcriptional regulators promoting differentiation into one of these lineages actively suppress differentiation into the other lineage [8]. It has therefore been postulated that a reciprocal relationship exists between the osteogenic and adipogenic pathways, implying that impaired adipogenic differentiation of hMSCs may result in enhanced osteogenic differentiation [3].

Multiple regulators are known that affect the choice between the osteogenic and adipogenic lineage. Most notably, activation of the WNT/β-catenin pathway promotes osteogenic differentiation and inhibits adipogenic differentiation of hMSCs [9]. BMP enhances the outgrowth of both osteoblasts and adipocytes, while the related cytokine transforming growth factor beta (TGFβ) inhibits both osteogenic and adipogenic differentiation, at least when tested under these in-vitro conditions [10, 11]. Moreover, Kim et al. [12] have shown that cAMP-activated protein kinases regulate the differentiation choice of hMSCs between osteogenesis and adipogenesis.

In order to find drugs affecting the choice between osteogenic and adipogenic differentiation of hMSCs, we have optimized the adipogenic culture conditions in such a way that, within the same culture, a fraction of the cells differentiates into osteoblasts and another fraction into adipocytes. Our results show that addition of TGFβ to these cultures fully blocks adipogenic differentiation but, surprisingly, enhances osteogenic differentiation. Analysis of the individual components in the culture medium showed that the presence of the phosphodiesterase inhibitor IBMX, which stabilizes cAMP levels in the cell, converted TGFβ from an inhibitor to an enhancer of osteogenic differentiation.

Based on these observations we have set up a gene expression microarray experiment to identity genes that under these optimized adipogenic culture conditions are downregulated by TGFβ. The genes identified in this way seem to play an important role in adipogenesis, since inhibitors of the corresponding proteins were found to be able to block adipogenic differentiation of hMSCs. Some of these inhibitors have already received FDA approval for the treatment of various diseases, and may therefore be good candidates for therapeutic drug repurposing in order to treat patients suffering from such diseases as obesity and osteoporosis.

## Methods

### Culture and differentiation of hMSCs

hMSCs harvested from normal human bone marrow were purchased from Lonza (Walkersville, MD, USA) at passage 2. Cells were expanded for no more than five passages in “mesenchymal stem cell growth medium” (MSCGM; Lonza) at 37 °C in a humidified atmosphere containing 7.5 % CO_2_. Studies were performed with hMSCs from three different donors, encoded 5F0138, 6F4085, and 7F3458.

For differentiation experiments, 4.0 × 10^4^ cells per cm^2^ were seeded in high-glucose-containing Dulbecco’s modified Eagle’s medium (DMEM) supplemented with 10 % fetal bovine serum (a selected lot from Lonza), 100 U/ml penicillin, and 100 μg/ml streptomycin. This medium will be further referred to as “proliferation medium” (PM). The next day, cells were switched to either osteogenic or adipogenic differentiation medium. Cells treated with PM were used as negative controls. Osteogenic differentiation medium (ODM) was composed of PM supplemented with 10^−7^ M DEX, 0.2 mM ascorbate, and 10 mM β-glycerophosphate. Adipogenic differentiation medium (ADM) was composed of PM supplemented with 10^−6^ M DEX, 10 μg/ml insulin (R&D Systems, Minneapolis, MN, USA), 10^−7^ M rosiglitazone (Sigma-Aldrich, St. Louis, MO, USA), and 500 μM IBMX (Sigma-Aldrich). Unless indicated otherwise, recombinant human TGFβ1 and BMP2 (both from R&D Systems) were used at concentrations of 2 ng/ml and 125 ng/ml, respectively. Prostaglandin E2 (PGE2), Batimastat, and Zopolrestat (Sigma-Aldrich) were used at the indicated concentrations. Media were refreshed every 3–4 days.

### Alkaline phosphatase assays

To quantify alkaline phosphatase (ALP) enzymatic activity as a measure of osteogenic differentiation, hMSCs were seeded in 96-well tissue culture plates as already described, after which cells were differentiated for 7 days in osteogenic differentiation medium. ALP enzymatic activity was quantified by measuring the formation of *p*-nitrophenol from *p*-nitrophenyl phosphate (PNPP; Sigma-Aldrich), as described previously [13]. ALP enzymatic activity was corrected for differences in cell number, as determined by a Neutral Red assay. Cells were incubated with Neutral Red dye diluted in PBS for 1 hour at 37 °C. After washing with PBS, the dye was extracted from the cells using 0.05 M NaH_2_PO_4_ in 50 % EtOH, after which the absorbance was measured at 540 nm.

For histochemical analysis of ALP activity, cells were seeded in 48-well tissue culture plates and differentiated for 7 days in osteogenic differentiation medium. Subsequently, cells were fixed in 3.7 % formaldehyde/PBS for 10 min at 22 °C. After washing with PBS, cells were incubated for 1 hour at 37 °C in a mixture of 0.1 mg/ml naphtol AS-MX phosphate (Sigma-Aldrich), 0.5 % *N*,*N*-dimethylformamide, 2 mM MgCl_2_, and 0.6 mg/ml Fast Blue BB salt (Sigma-Aldrich) in 0.1 M Tris–HCl, pH 8.5.

### Mineralization assay

To measure calcium deposition in the extracellular matrix, hMSCs were seeded in 24-well tissue culture plates and cultured for 13 days under osteogenic or adipogenic differentiation conditions, as indicated. Cells were subsequently washed twice with PBS after which calcium was extracted from the extracellular matrix by treatment with 150 μl of 0.5 M HCl. Calcium concentrations were measured in a colorimetric assay using *o*-cresolphtalein complexone as a chromogenic agent, according to the protocol provided by the manufacturer (Sigma-Aldrich).

### Oil Red O staining and Triglyceride assay

To quantify adipogenic differentiation, lipid droplets were stained in mature adipocytes obtained after treatment of hMSCs for 9 days in adipogenic differentiation medium. Cells were first washed twice with PBS, fixed for 30 min with 1 % formaldehyde in PBS, and then washed once with water and twice with 60 % isopropanol. Cells were then stained for 1 hour with 0.3 % w/v Oil Red O (Sigma-Aldrich) in 60 % isopropanol. Subsequently, cells were washed once with 60 % isopropanol and twice with distilled water. For quantification of Oil Red O staining, samples were treated with 100 % isopropanol and absorbance was measured at 530 nm.

The amount of triglycerides stored in lipid droplets of mature adipocytes was quantified after treatment of hMSCs for 9 days in adipogenic differentiation medium. Cells in 96-well tissue culture plates were washed twice with PBS. Triglycerides were extracted from the lipid droplets by freezing the cells in 50 μl of a buffer containing 25 mM Tris–HCl (pH 7.5) and 1 mM EDTA, followed by addition of 40 μl *tert*-butanol and 10 μl methanol. Samples were heat-dried at 55 °C, after which they were resuspended in Triglycerides LiquiColor® mono reagents (HUMAN GmbH, Wiesbaden, Germany). Triglycerides were quantified by measuring the absorbance at 490 nm.

### RNA isolation and real-time quantitative RT-PCR

RNA was isolated as described by Piek et al. [13]. For cDNA synthesis, 1 μg of total RNA was reverse transcribed using random hexamer primers and SUPERSCRIPT™ II reverse transcriptase (Invitrogen, Carlsbad, CA, USA). Subsequently, cDNA was amplified in a quantitative real-time PCR, performed using Power SYBR Green® PCR Mastermix (Applied Biosystems, Foster City, CA, USA) on an Applied Biosystems 7500 Real-time Fast PCR System. For each gene, PCR was carried out in duplicate and mean expression values were calculated relative to the mean expression level of the housekeeping gene *RPS27A* (ribosomal protein S27a). Human gene-specific PCR primers used included the following:

- Histone deacetylase 5 (*HDAC5*)-FW: 5′-ATGACAACGGGAACTTCTTTCC-3′.
- Histone deacetylase 5 (*HDAC5*)-RV: 5′-CCATGCCACGTTCACATTGTA-3′.
- Adiponectin (*ADIPOQ*)-FW: 5′-CCCAAAGAGGAGAGAGGAAGC-3′.
- Adiponectin (*ADIPOQ*)-RV: 5′-GCCAGAGCAATGAGATGCAA-3′.
- Alkaline phosphatase (*ALPL*)-FW: 5′-GATGGACAAGTTCCCCTTCGT-3′.
- Alkaline phosphatase (*ALPL*)-RV: 5′-GGACCTGGGCATTGGTGTT-3′.
- Ribosomal protein S27a (*RPS27A*)-FW: 5′-GTTAAGCTGGCTGTCCTGAAA-3′.
- Ribosomal protein S27a (*RPS27A*)-RV: 5′-CATCAGAAGGGCACTCTCG-3′.

### Immunoblotting

hMSCs were seeded at 4.0 × 10^4^ cells per cm^2^ in six-well plates and cultured for 24 hours in the indicated differentiation media. Cells were then lysed in 250 μl of RIPA lysis buffer per well. Then 5 μl of reducing sample buffer was added to 25 μl of lysate, heated to 95 °C, and subsequently loaded onto an 8 % SDS-PAGE gel. HDAC5 was detected on blots using a goat polyclonal anti-HDAC5 antibody (G-18, 1/200 dilution) raised against the N-terminus of human HDAC5 (sc-5250; Santa Cruz Biotechnology, Dallas, TX, USA), followed by an HRP-labeled polyclonal secondary antibody. Antibodies against α-tubulin (Sigma) served as a loading control.

### Gene expression microarray analysis

To identify genes that are regulated during osteogenic and adipogenic differentiation of hMSCs, a total of 54 samples (each containing 800,000 cells/ 20 cm^2^) were seeded in PM and grown for 24 hours. Subsequently the medium was exchanged for differentiation medium, now consisting of PM with 10^−6^ M DEX, 10 μg/ml insulin, 10^−7^ M rosiglitazone, and 50 ng/ml BMP2 (B). In addition, either 5 ng/ml TGFβ (BT), or 250 μM IBMX (BI), or 5 ng/ml TGFβ and 250 μM IBMX (BTI) were added. Samples were incubated for either 0, 1, 2, 3, or 7 days. Experiments for each group and time point were carried out as three biological replicates, while the untreated control group (time 0) consisted of six samples. RNA was isolated as already described, and hybridized onto Affymetrix HGU 133 plus 2.0 microarrays according to existing protocols [13].

Microarray data were analyzed with the R language for statistical computing using appropriate Bioconductor packages (http://bioconductor.org/) for reading, normalizing, and statistically evaluating the data, followed by annotation of the gene sets and integration of parallel data sources. Briefly, the analysis started with a careful quality assessment of the dataset using the automatic R pipeline AffymetrixQC [14], which was customized and run locally. All 54 microarrays passed the quality control and were included in the analysis, consisting of robust microarray analysis (RMA) normalization [15], followed by statistical analysis to find differentially expressed genes using Linear Models for Microarray Data (LIMMA) [16], and subsequent functional annotation and enrichment analysis using the online resource Database for Annotation, Visualization and Integrated Discovery (DAVID) [17, 18]. Finally, the list of differentially expressed genes for the contrasts of interest was crossed with the information from the DrugBank database [19] in order to derive the final list of candidate genes for experimental testing. All R scripts used for this analysis are available upon request. Current microarray data have been deposited in NCBI’s Gene Expression Omnibus [GEO:GSE84500] (https://www.ncbi.nlm.nih.gov/geo/query/acc.cgi?acc=GSE84500).

### Statistical analysis

Student’s *t* test was used for statistical comparisons. Numeric data are represented as mean ± standard deviation of triplicate experiments, unless stated otherwise.

## Results

### TGFβ induces hMSCs to switch from adipogenic to osteogenic differentiation

**Fig. 1:**
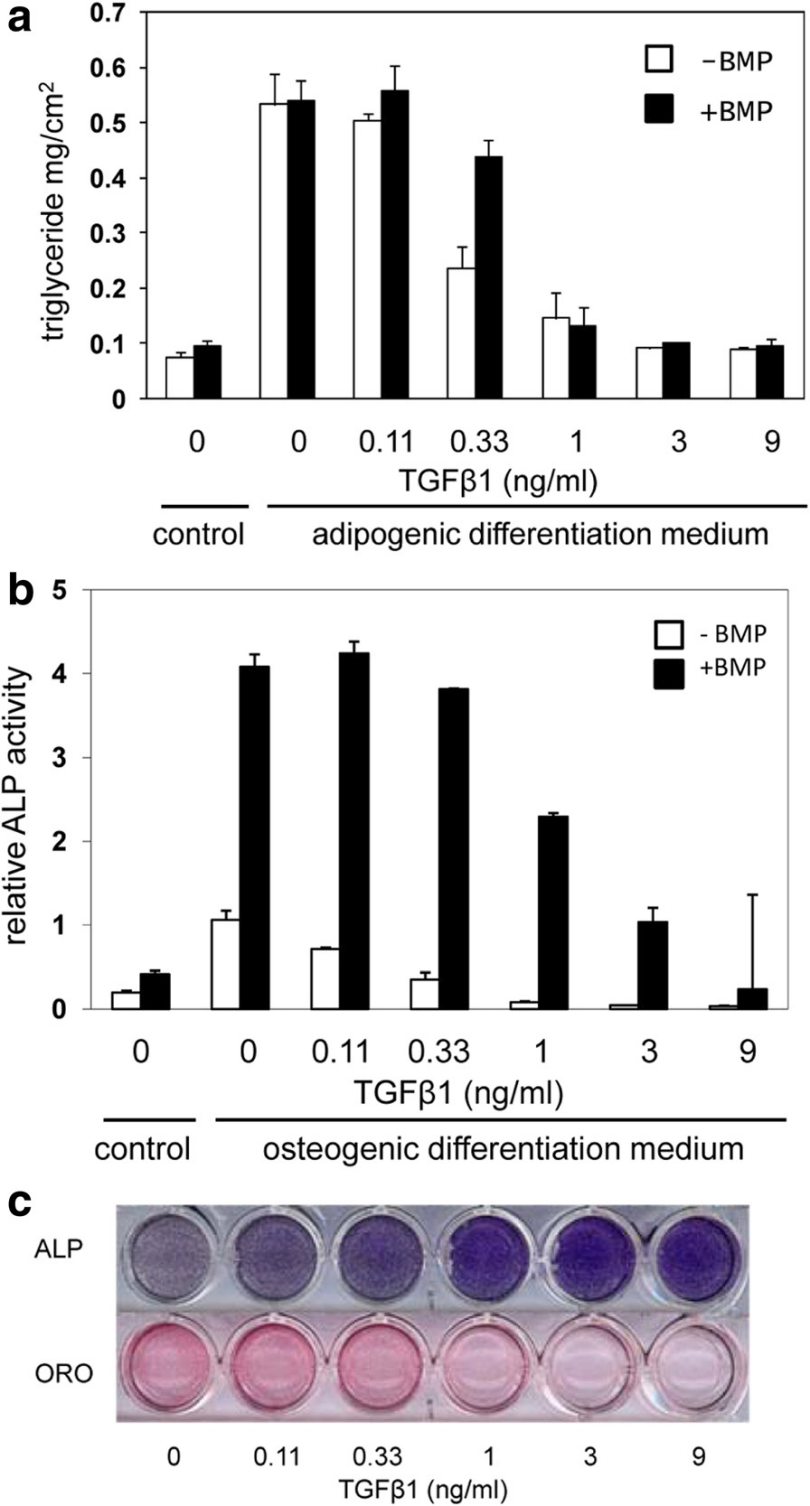
Effect of TGFβ on adipogenic and osteogenic differentiation of hMSCs. **a** Triglyceride production by hMSCs, 9 days after incubation with adipogenic differentiation medium in the absence (*white shading*) and presence (*black shading*) of 125 ng/ml BMP2 and increasing concentrations of TGFβ1. The enhancing effect of BMP2 is not significant, while the inhibitory effect of TGFβ is significant (*p* < 0.01) above 1 ng/ml. **b** Alkaline phosphatase (*ALP*) activity of hMSCs, 7 days after incubation with osteogenic differentiation medium in the absence (*white shading*) and presence (*black shading*) of 125 ng/ml BMP2 and increasing concentrations of TGFβ1. Enhancing effect of BMP2 is significant (*p* < 0.01) at all data points, while the inhibitory effect of TGFβ is significant (*p* < 0.01) above 1 ng/ml. **c** TGFβ-induced switch from adipogenic to osteogenic differentiation. ALP staining (after 7 days) and Oil Red O (*ORO*) staining (after 9 days) following incubation of hMSCs in adipogenic differentiation medium containing 125 ng/ml BMP2 and the indicated concentrations of TGFβ1. *BMP* bone morphogenetic protein, *TGFβ* transforming growth factor beta

BMPs have been described as positive regulators of both osteogenesis and adipogenesis [8, 10]. In order to study the effect of BMP2 on differentiation of hMSCs in more detail, we cultured these cells in either osteogenic differentiation medium or adipogenic differentiation medium in the absence and presence of BMP2. Figure 1a shows that addition of BMP2 has only a small stimulatory effect on adipogenic differentiation of hMSCs, as measured by the amount of the triglyceride production. On the other hand, BMP2 strongly enhanced osteogenic differentiation, as indicated by increased ALP activity (Fig. 1b).

The role of TGFβ in adipogenic and osteogenic differentiation of hMSCs is still unclear. Figure 1a also shows that adding TGFβ to adipogenic differentiation medium blocks adipogenic differentiation of hMSCs in a dose-dependent manner, both in the absence and additional presence of BMP2. Figure 1b shows that addition of TGFβ to osteogenic differentiation medium results in a similar inhibition of osteogenic differentiation, both in agreement with previous data [10, 11]. However, when hMSCs are treated with adipogenic differentiation medium (which contains a 10-fold higher concentration of DEX than osteogenic differentiation medium) in combination with BMP2, a fraction of the cells will differentiate into bone cells and a fraction into fat cells within the same well, as shown in Fig. 1c by histological staining. Addition of TGFβ under these conditions resulted is a dose-dependent increase in the number of ALP-positive bone cells, with a concomitant reduction in Oil Red O-positive fat cells. These data show that TGFβ blocks bone cell differentiation under osteogenic differentiation conditions, but enhances bone cell differentiation under adipogenic differentiation conditions. It can therefore be concluded that, under the experimental conditions used in Fig. 1c, TGFβ induces a switch from adipogenic to osteogenic differentiation.

### IBMX is a critical component in the TGFβ-mediated switch in cell fate

**Fig. 2:**
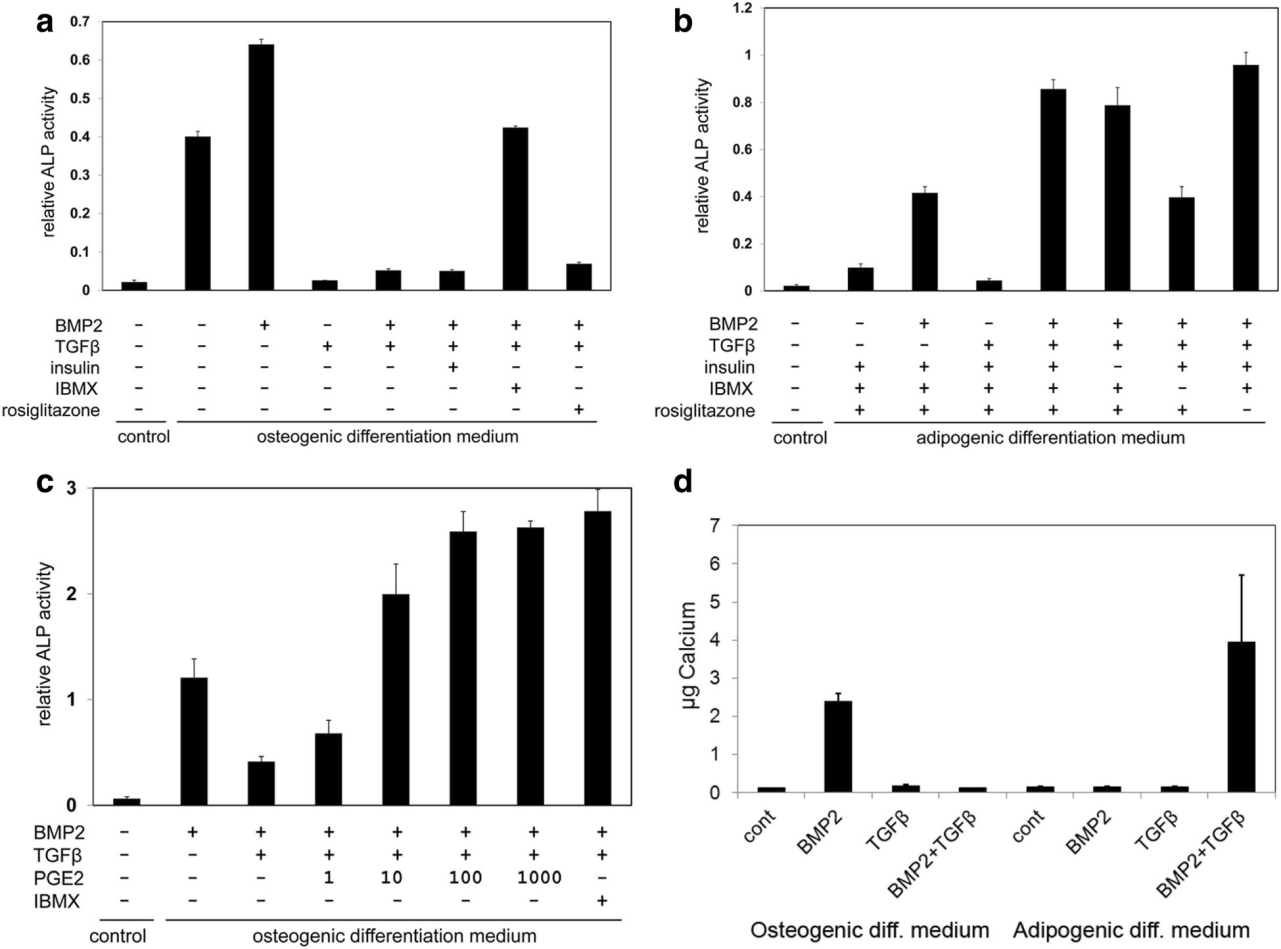
cAMP regulators control TGFβ-induced osteogenic differentiation of hMSCs. Osteogenic differentiation was measured by ALP activity and corrected for the level of Neutral Red uptake as a measure for the number of cells present in the well. **a** Osteogenic differentiation of hMSCs in osteogenic differentiation medium supplemented with or without 125 ng/ml BMP2, 2 ng/ml TGFβ1, 10 μg/ml insulin, 500 μM IBMX, or 10^−7^ M rosiglitazone. ALP activity is significantly higher (*p* < 0.001) in medium with BMP2 and TGFβ in the presence of IBMX than in the absence of IBMX. **b** Osteogenic differentiation of hMSCs in adipogenic differentiation medium supplemented with or without 125 ng/ml BMP2 and 2 ng/ml TGFβ1, and following omission of 10 μg/ml insulin, 500 μM IBMX, or 10^−7^ M rosiglitazone. ALP activity is significantly higher (*p* < 0.01) in medium with all supplements than in the absence of IBMX. **c** Effect of PGE2, added at the indicated nanomolar concentrations, on osteogenic differentiation of hMSCs in osteogenic differentiation medium. A comparison is made with the effects of BMP2 (125 ng/ml), TGFβ (2 ng/ml), and IBMX (500 μM). Enhancement of ALP activity is significant (*p* < 0.05) at concentrations of 10 nM PGE2 and above. **d** Effect of BMP2 (125 ng/ml) and TGFβ (2 ng/ml) on total Ca^2+^ deposition (μg) in a six-well plate well (10 cm^2^) by hMSCs, cultured for 13 days in either osteogenic or adipogenic differentiation medium. Ca^2+^ deposition is significantly enhanced by BMP2 alone in osteogenic differentiation medium (*p* < 0.01) and by BMP2 + TGFβ in adipogenic differentiation medium (*p* < 0.05). *ALP* alkaline phosphatase, *BMP* bone morphogenetic protein, *IBMX* 3-isobutyl-1-methylxanthine, *PGE2* prostaglandin E2, *TGFβ* transforming growth factor beta

In order to investigate which component of the adipogenic differentiation medium allows osteogenic differentiation in the presence of TGFβ, we added the adipogenic differentiation medium components insulin, IBMX, and rosiglitazone successively to hMSCs grown in osteogenic differentiation medium with BMP2 and TGFβ. Figure 2a shows that the inhibition of osteogenic differentiation by TGFβ could not be prevented by insulin or rosiglitazone, but was largely overcome upon addition of IBMX. A parallel experiment showed that omission of IBMX from adipogenic differentiation medium was sufficient to prevent the TGFβ-induced enhancement of osteogenic differentiation, while removal of insulin or rosiglitazone was without effect (Fig. 2b).

IBMX is a phosphodiesterase inhibitor, which prevents degradation of cAMP. In order to show that enhanced cAMP levels play a role in the TGFβ-mediated switch in cell fate, we tested the effect of PGE2, a known activator of adenylate cyclase. Figure 2c shows that PGE2 is able to overcome TGFβ-induced inhibition of osteogenic differentiation of hMSCs in a dose-dependent manner, to a similar extent as IBMX.

Osteogenic differentiation requires not only matrix maturation, as indicated by alkaline phosphatase expression, but also matrix mineralization, as indicated by calcium deposition. Figure 2d shows that under osteogenic differentiation conditions BMP is required for calcium deposition by hMSCs, while both BMP and TGFβ are required under adipogenic differentiation. These data show TGFβ is able to induce fully differentiated, mineralized osteoblasts under adipogenic differentiation conditions.

### IBMX suppresses HDAC5 expression

Previous studies [20] have shown that TGFβ-mediated inhibition of osteogenic differentiation can be overcome by trichostatin A, an inhibitor of class I and II mammalian histone deacetylases (HDACs). We confirmed this observation upon incubating hMSCs in osteogenic differentiation medium with BMP2 and TGFβ (data not shown). Kang et al. [20] have presented evidence that HDAC4/5 can interact with TGFβ-activated SMAD3, resulting in a complex that represses transcription of the bone marker gene osteocalcin (BGLAP). In human adipose-derived mesenchymal stem cells, trichostatin A impaired PPARG activity, at least under osteogenic differentiation conditions [21]. Still, the role of HDACs in adipogenesis is far from clear, since HDAC inhibitors may either enhance or inhibit adipogenic differentiation [22].

**Fig. 3:**
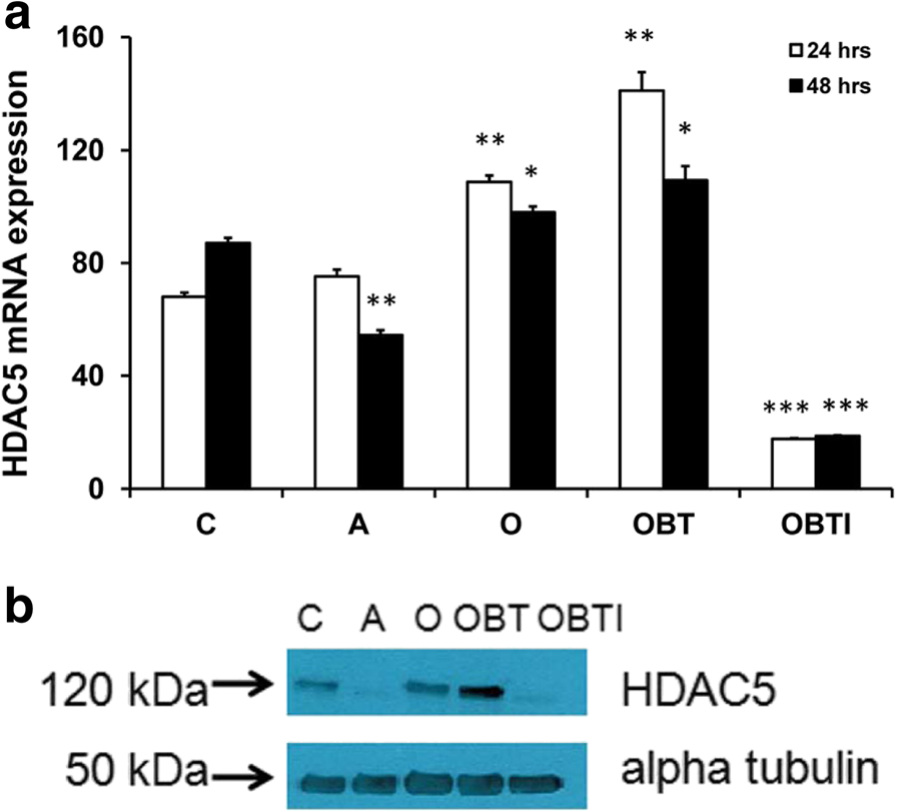
IBMX controls HDAC5 expression levels in hMSCs. **a** HDAC5 mRNA levels, relative to that of the housekeeping gene *RSP27A*, of hMSCs incubated for 24 hours (*white shading*) or 48 hours (*black shading*) in proliferation medium (*C*), adipogenic differentiation medium (*A*), osteogenic differentiation medium (*O*), osteogenic differentiation medium supplemented with 125 ng/ml BMP2 and 2 ng/ml TGFβ (*OBT*), and osteogenic differentiation medium supplemented with 125 ng/ml BMP2, 2 ng/ml TGFβ, and 500 μM IBMX (*OBTI*). Data expressed as percentage of the expression levels at time zero. Results represent the mean and standard deviation of duplicate experiments. Indicated significance levels are relative to expression in control samples (C) at 24 or 48 hours, respectively. **b** Immunoblot for protein expression of HDAC5 in hMSCs, 5 days after incubation in the indicated media. Expression of α-tubulin was used as a loading control. *p < 0.05; **p < 0.01; ***p < 0.001. *HDAC* histone deacetylase

We have studied the expression of HDAC5 under conditions that TGFβ enhances osteogenic differentiation of hMSCs. Figure 3a shows that HDAC5 mRNA levels are slightly upregulated 24–48 hours after addition of osteogenic differentiation medium, particularly in the presence of BMP2 and TGFβ (OBT). A strong reduction in HDAC5 gene expression was observed when IBMX was added to the medium (OBTI), however, thus creating conditions under which adipogenic differentiation is prevented and osteogenic differentiation is promoted. Less reduction in HDAC5 mRNA levels was observed in adipogenic differentiation medium alone. We could not detect mRNA expression of HDAC4 in these cells.

Figure 3b shows that HDAC5 levels are similarly regulated at the protein level. Western blot analysis revealed the highest expression level in hMSCs treated with osteogenic differentiation medium containing BMP2 and TGFβ (OBT), whereas almost no protein was detected in the additional presence of IBMX (OBTI). These data indicate that under conditions whereby TGFβ enhances osteogenic differentiation, no HDAC5 is available to prevent expression of bone specific genes.

### Altered gene expression during TGFβ-mediated switch in cell fate

**Fig. 4:**
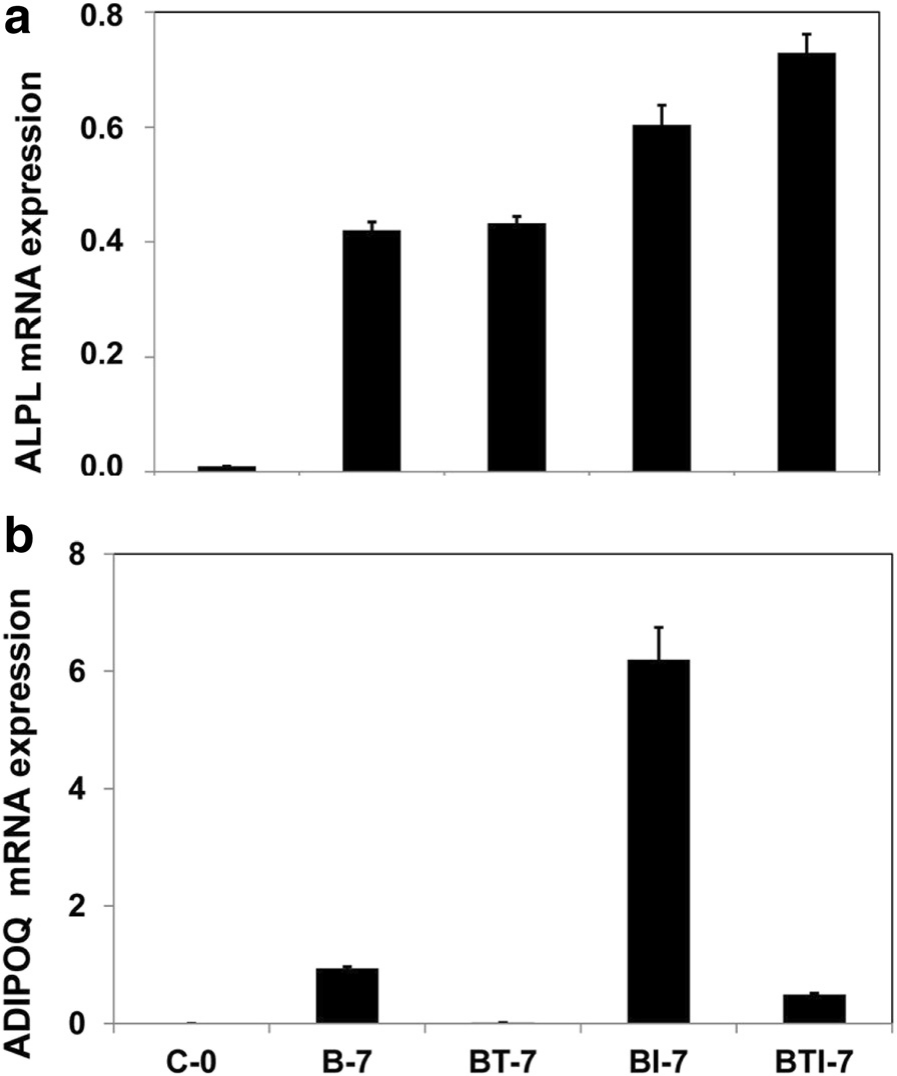
Expression of osteogenic and adipogenic markers in hMSCs under conditions used for gene expression microarray analysis. Cells were incubated for 7 days in adipogenic differentiation medium containing 50 ng/ml BMP2 (*B-7*), 50 ng/ml BMP2 + 5 ng/ml TGFβ (*BT-7*), 50 ng/ml BMP2 + 500 μM IBMX (*BI-7*), and 50 ng/ml BMP2 + 5 ng/ml TGFβ + 500 μM IBMX (*BTI-7*). Untreated cells at day zero (*C-0*) were used as a control. **a** mRNA expression of the bone marker ALP (*ALPL*), relative to that of the housekeeping gene *RSP27A*. Results represent the mean and standard deviation of duplicate experiments. Both BI-7 (*p* < 0.05) and BTI-7 (*p* < 0.01) are significantly higher than B-7 and BT-7. **b** mRNA expression of the fat marker adiponectin (*ADIPOQ*), relative to that of the housekeeping gene *RSP27A*. Results represent the mean and standard deviation of duplicate experiments. BI-7 is significantly higher than both B-7 and BTI-7 (*p* < 0.01)

The presented data show that, upon incubation of hMSCs in adipogenic differentiation medium containing BMP2 and IBMX, a fraction of the cells will differentiate into ALP-positive bone cells and a fraction into Oil Red O-positive fat cells. Subsequent addition of TGFβ reduces the number of fat cells and enhances the number of bone cells in a dose-dependent manner (see Fig. 1c). This observation implies that under these experimental conditions addition of TGFβ will stimulate the expression of osteoblast genes and reduce the expression of adipocyte genes. In order to identify genes involved in this lineage switch, we performed gene expression microarray analysis on hMSCs, treated for 1, 2, 3, or 7 days with differentiation medium containing DEX, insulin, and rosiglitazone, using as supplements BMP2 and combinations of IBMX and TGFβ. Untreated cells at day 0 served as the control experiment. In order to verify the extent of differentiation of the cells used for the microarray experiments, we used real-time PCR to measure mRNA expression levels of the osteoblast-specific alkaline phosphatase (*ALPL*) gene and of the adipocyte-specific adiponectin (*ADIPOQ*) gene, a target gene of the adipogenic master gene *PPARG*. This analysis was carried out 7 days after incubation with BMP2 (B), BMP2 + TGFβ (BT), BMP2 + IBMX (BI), or BMP2 + TGFβ + IBMX (BTI). Figure 4a shows that under these conditions IBMX, in combination with TGFβ, enhanced *ALPL* expression. No inhibitory effect of TGFβ was observed on bone cell differentiation, in agreement with the data of Fig. 2b. *ADIPOQ* expression was strongly enhanced upon IBMX addition, but reduced again to very low levels in the additional presence of TGFβ (Fig. 4b). These data confirm that, at the gene expression level, TGFβ induces a switch from adipocytes to osteoblasts.

**Fig. 5:**
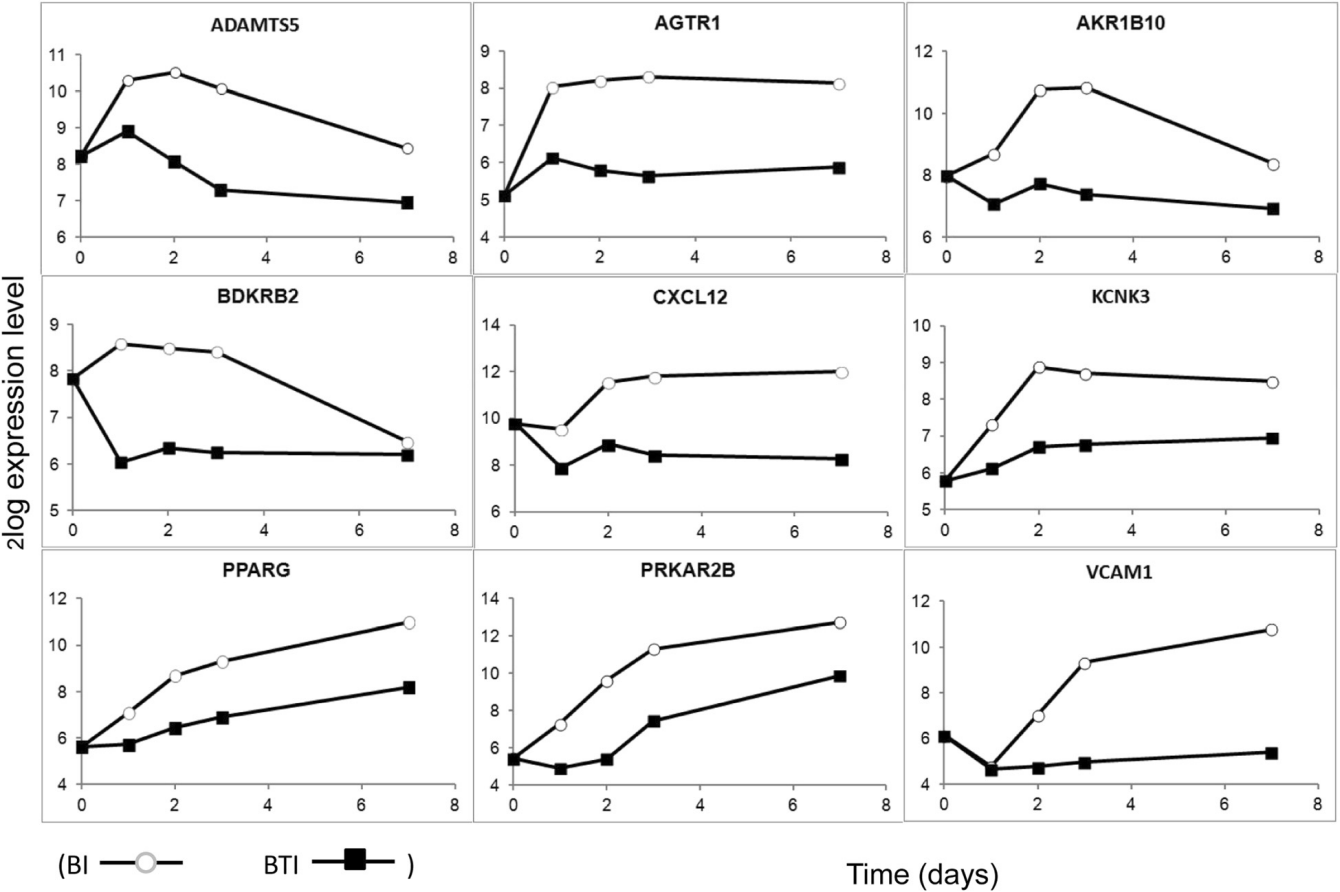
Time course for expression of the nine selected genes for their involvement in adipogenic differentiation. Expression levels were obtained from analysis of the 50 ng/ml BMP2 and 250 μM IBMX (*BI*, *white circle*) and 50 ng/ml BMP2, 5 ng/ml TGFβ, and 250 μM IBMX (*BTI*, *black square*) chips. Expression levels are indicated on a log_2_ scale. *BMP* bone morphogenetic protein, *IBMX* 3-isobutyl-1-methylxanthine, *TGFβ* transforming growth factor beta

The data from the 54 microarray samples were normalized using RMA [15] followed by LIMMA [16] statistical analysis to identify differentially expressed genes and functional enrichment analysis using DAVID [17, 18]. From the 54,675 probes on the chip, 7755 probes appeared differentially expressed at any time point or treatment, compared with the *t* = 0 control, based on a *q* value of 10^−5^ and a minimum log_2_-fold change of 1. Our primary interest was to identify genes that controlled the TGFβ-induced switch from adipogenic to osteogenic differentiation. In the current experiment, genes downregulated by TGFβ are potentially involved in adipogenic differentiation and genes upregulated by TGFβ in osteogenic differentiation. A comparison between the samples BTI and BI resulted in 2911 differentially expressed probes at any time point, of which 1176 probes (735 genes) were differentially expressed at the early time points (days 1 and 2), when cells become committed for lineage specific differentiation. Extending the log_2_-fold change from 1 to a minimum of 2 resulted in a reduction of the number of differentially expressed genes between BTI and BI to 109, of which 25 were established drug targets according to the DrugBank database (www.drugbank.ca). Visual inspection of the time course for expression of these genes identified nine genes which showed the desired dynamics; that is, modulation at early time points and higher expression in BI than in BTI, as presented in Fig. 5.

### Analysis of adipogenic differentiation inhibitors

For the thus identified target genes, as presented in Table 1, we tested whether commercially available inhibitors could block the adipogenic differentiation of hMSCs under conditions similar to those used for the microarray analysis. We first confirmed the established observation that adipogenic differentiation does not occur in the absence of a PPARG agonist, such as rosiglitazone, and therefore an antagonist of this receptor was not further tested. We did, however, also observe that inhibitors of ADAMTS5 and AKR1B10 prevented adipogenic differentiation of hMSCs, while inhibitors of AGTR1, BDKRB2, and KCNK3 were without effect. Earlier studies in the literature have already indicated that inhibitors of metalloproteinases, including Batimastat, have a negative influence on adipogenic differentiation [23], but this is the first report for an inhibitory effect of an aldo-keto reductase inhibitor.

**Table 1:** Selected genes involved in adipogenic differentiation

Gene name	Protein function	Inhibitor	Effect
*ADAMTS5*	Metalloproteinase	Batimastat	Positive
*AGTR1*	Angiotensin II receptor	Losartan	Negative
*AKR1B10*	Aldo-keto reductase	Zopolrestat	Positive
*BDKRB2*	Bradykinin receptor B2	Icatibant	Negative
*CXCL12*	Chemokine SDF-1	(Not tested)	(Not tested)
*KCNK3*	Potassium channel	Doxapram	Negative
*PPARG*	Nuclear receptor	GW9662	Positive
*PRKAR2B*	cAMP protein kinase	(Not tested)	(Not tested)
*VCAM1*	Vascular cell adhesion	(Not tested)	(Not tested)

Table presents the HUGO name, function, commercially available drug, and status of experimentally observed effects

*positive* observed effect, *negative* no observed effect

**Fig. 6:**
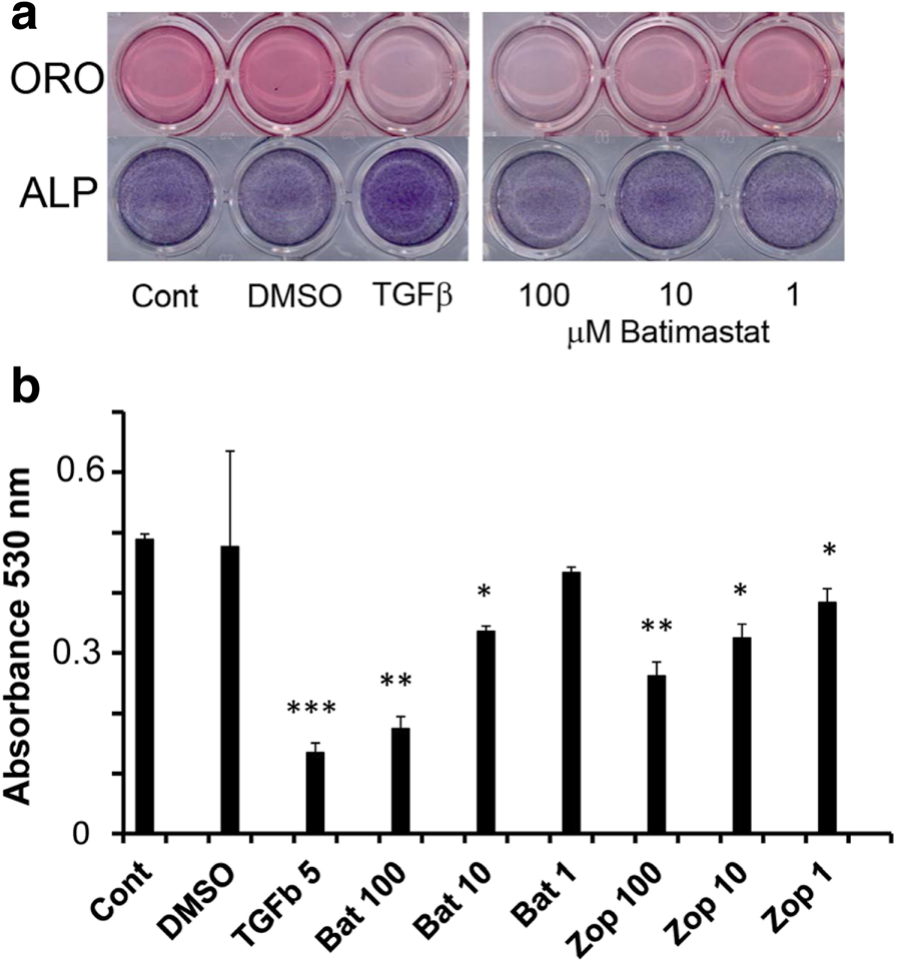
Effect of selected inhibitors on adipogenic differentiation of hMSCs. **a** Effect of Batimastat, added at the indicated concentrations to hMSCs in adipogenic differentiation medium, on adipogenic (*upper panel*; Oil Red O (*ORO*) staining, day 9) and osteogenic differentiation (*lower panel*; alkaline phosphatase (*ALP*) staining, day 7). No addition (*Cont*), TGFβ (2 ng/ml), and DMSO (0.1 %; used as a solvent for Batimastat) were used as controls. **b** Inhibition of adipogenic differentiation by Batimastat (*Bat*) and Zopolrestat (*Zop*), added at the indicated micromolar concentrations. Quantitative Oil Red O staining was measured at 530 nm on hMSCs treated for 9 days with adipogenic differentiation medium. TGFβ (5 ng/ml) was used as a control. Significance levels are indicated relative to untreated controls (*Cont*). *p < 0.05; **p < 0.01; ***p < 0.001. *DMSO* dimethyl sulfoxide, *TGFβ* transforming growth factor beta

Figure 6a shows the effect of Batimastat on adipogenic and osteogenic differentiation of hMSCs under the experimental conditions used for the microarray analysis. TGFβ and DMSO, as a vehicle for Batimastat, were used as a positive and a negative control. The data show that Batimastat inhibits adipogenic differentiation in a dose-dependent manner but, in contrast to TGFβ, this inhibition does not result in a concomitant enhancement of osteogenic differentiation. A similar observation was made for the AKR1B10 inhibitors Sorbinil (not shown) and Zopolrestat. The quantitative analysis presented in Fig. 6b shows that Zopolrestat actively inhibits adipogenic differentiation of hMSCs in a dose-dependent manner, although to a lesser extent than Batimastat.

## Discussion

Osteoporosis is a debilitating disease which affects tens of millions of people worldwide. Postmenopausal women are particularly at risk of developing this disease, since reduced estrogen activity enhances the bone-resorbing activity of osteoclasts. Additionally, aging in general results in a significant reduction in the number and function of bone-forming osteoblasts [24]. Treatment of patients with osteoporosis may include hormone replacement therapies and the use of bisphosphonates to inhibit the activity of osteoclasts. Moreover, vitamin D3 or parathyroid hormone can be prescribed to enhance new bone formation [25]. Recent years have seen an increasing interest in the interaction between fat and bone cells in the bone marrow. Patients suffering from osteoporosis show an increased number of adipocytes in their bone marrow, concomitant with a reduction in the pool of hMSCs that are able to differentiate into osteoblasts [2, 3]. In addition, adipokines secreted by these adipocytes may further enhance osteoclast activity [26], while an increasing fat content of the bone marrow may also impair the bone cell niche required for the functioning of hematopoietic stem cells [27].

These considerations show that there is great need for developing drugs that prevent the differentiation of hMSCs into fat cells and may thereby enhance their differentiation into bone cells. Likewise, drugs that prevent adipogenic differentiation could also be useful in the battle against obesity. In the present study we have shown that, under proper in-vitro conditions, TGFβ is able to stimulate osteogenic differentiation and prevent adipogenic differentiation within the same culture. These culture conditions require not only the presence of DEX and BMP, but also of a cAMP-enhancing stimulus such as IBMX or PGE2. Because of its strong side effects TGFβ is not suited for in-vivo applications, but our results show that the current in-vitro system can be used for identifying genes whose expression is repressed following a TGFβ-induced switch from adipogenic to osteogenic differentiation of hMSCs. By concentrating on those genes for which FDA-approved drugs are available, we have identified nine potential genes that could be tested for inhibition of adipogenic differentiation of hMSCs. Using this approach we have identified the nuclear hormone receptor PPARG, the metalloproteinase ADAMTS5, and the aldo-keto reductase AKR1B10 as potential drug targets for treatment of osteoporosis and obesity.

TGFβ is a highly pleiotropic cytokine which plays an important role in many physiological processes, as well as in cancer [28, 29]. Because of its strong inhibitory effect on the immune system, systemic treatment with TGFβ is not considered a realistic option, except for protection against autoimmune diseases [30]. Studies in laboratory animals have shown that TGFβ treatment can result in skin fibrosis and toxicity, without displaying significant antitumor effects [31, 32]. Local injection of TGFβ into the knee joint has been shown to enhance cartilage integrity, leading to prevention of osteoarthritis [33]. Our present study shows that the effect of TGFβ on osteogenic differentiation of hMSCs strongly depends on the culture conditions. In osteogenic differentiation medium TGFβ inhibits bone cell differentiation, while it promotes this process in adipogenic differentiation medium. Since the local conditions in the fatty bone marrow of osteoporosis patients are not well defined, it is difficult to predict whether injection of TGFβ will result in a net enhancement or a decrease of functional osteoblast cells. Furthermore, current clinical trials directed towards TGFβ are aimed at inhibiting its activity in cancer patients, since overactivation of TGFβ-induced pathways have been associated with cancer progression [28, 29].

Among the genes that were downregulated at early time points following TGFβ treatment of hMSCs in adipogenic differentiation medium is *PPARG*. This observation is not unexpected, since ligand-induced activation of PPARG (e.g., by rosiglitazone) is known to be essential for adipogenic differentiation. We have studied the role of PPARG in this process not by using the PPARG-specific antagonist GW9662 (see Table 1), but by omitting rosiglitazone from the culture medium, which resulted in a complete block of fat cell formation (data not shown). The promoting role of PPARG agonists in obesity is well established, but on the contrary these hormones have been shown to be clinically active as antidiabetic drugs [34]. Because of these multiple faces of PPARG, inhibition of this nuclear hormone receptor does not seem an attractive approach for the prevention of fatty bone marrow formation in osteoporosis patients.

The second gene for which inhibitors were found to prevent fat cell differentiation is *ADAMTS5*, a disintegrin and metalloproteinase with thrombospondin motifs [35]. Its main function is the cleavage of the aggrecan core protein, in which it appears more efficient than other matrix metalloproteinases (MMPs) [36]. Recent studies on *Adamts5*^–/–^ mice, however, have indicated that *ADAMTS5* may not responsible for aggrecan proteolysis, but instead regulates glucose uptake by mediating the endocytotic trafficking of LRP1 and GLUT4 [37]. Other studies have shown that deletion of active *ADAMTS5* prevents cartilage degradation in a murine model of osteoarthritis [38]. Batimastat and the related drug Marimastat have primarily been developed as antineoplastic drugs. They prevent angiogenesis by blocking metalloproteinases including MMPs and ADAM family members [39]. Both drugs performed poorly in clinical trials on metastatic breast cancer patients [40] and were therefore never marketed. Previous studies have shown that Batimastat blocks the enzymatic activity of MMP2 and MMP9, and prevents the differentiation of mouse 3T3-F442A preadipocytes into fat cells [23, 41]. Our present results show that Batimastat also prevents adipogenic differentiation of hMSCs. Long-term studies on Batimastat and Marimastat in humans are lacking and therefore it cannot be concluded whether these drugs present a real potential for the treatment of patients with osteoporosis or obesity. MMPs are not only involved in cancer progression, but also play an important role in inflammation and immunity [42]. Multiple natural compounds have been identified which seem to block MMP activity [43]. It will be interesting to test the effects of these compounds, some of which are used as food supplements, in relation to osteoporosis and obesity.

In this study we have made the novel observation that inhibitors of aldo-keto reductases prevent adipogenic differentiation of hMSCs. *AKR1B10*, which was downregulated by TGFβ in our studies, is particularly active on lipid substrates. It plays an important role in the reduction of retinaldehyde to retinol, as well as in the lipid modification of the K-Ras oncogene. In humans, this gene is particularly expressed in the intestine, adrenal gland, and liver. Inhibition of AKR1B10 prevents the outgrowth of pancreatic carcinoma cells by modulating the Ras pathway [44, 45]. Moreover, expression of AKR1B10 is considered to be a tumor marker for NSCLC [46] and liver tumors [47]. To the best of our knowledge, a role of AKR1B10 in adipogenic differentiation has not yet been studied. However, an RNA-mediated knockdown study has indicated that AKR1B10 is an important regulator of fatty acid biosynthesis in human RAO-3 breast cancer cells [48].

*AKR1B10* is structurally very similar to *AKR1B1*, and many inhibitors of AKR1B1 also bind AKR1B10. These include the drugs Sorbinil and Zopolrestat, which have been used in the present study. These and related FDA-approved drugs are used particularly for treatment of patients with diabetic polyneuropathy [49]. They do so by inhibiting the metabolism of glucose by the so-called polyol pathway, which converts glucose into sorbitol. This reduced sugar accumulates in the cell and, as a result of osmotic stress, induces microvascular damage to the retina, kidney, and nerves. Although each of these drugs has its specific adverse effects, including rash, toxicity, and hypersensitivity reactions, at least some of them were well tolerated in clinical trials lasting more than a year [49]. However, no long-term beneficial effects of these drugs were observed in diabetes patients. More recently, AKR1B1 inhibitors have also been tested as anticancer drugs [50]. So far, no reports have been made in the literature on the effects of aldo-keto reductase inhibitors on patients with osteoporosis or obesity. Similar to observations for MMPs, multiple naturally occurring inhibitors for aldo-keto reductases have been identified, particularly from plant tissue [51, 52]. Feeding mice with the most potent of these natural AKR1B inhibitors, bisdemethoxycurcumin, has been shown to reduce particularly the incidence of intestinal cancer. Interestingly, some of these natural compounds inhibit not only AKR1B members but also MMPs. In ongoing research we are testing the effects of these natural compounds on fat cell differentiation. These studies may provide a lead towards the further development of more optimized compounds which specifically prevent adipogenic differentiation in vivo.

**Fig. 7:**
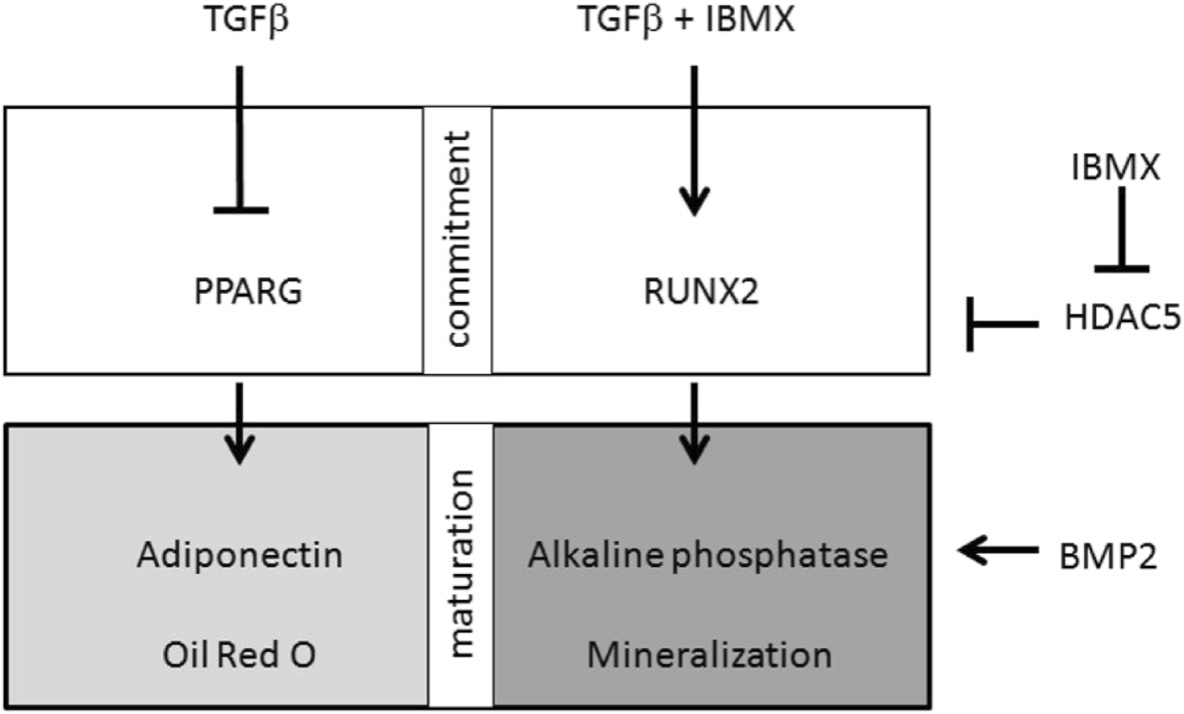
Overview of signaling pathways activated in hMSCs by TGFβ in the absence and presence of IBMX: adipogenic differentiation (*left*) and osteogenic differentiation (*right*). For details, see text. *BMP* bone morphogenetic protein, *HDAC* histone deacetylate, *IBMX* 3-isobutyl-1-methylxanthine, *TGFβ* transforming growth factor beta

TGFβ is generally considered to be an inhibitor of bone cell differentiation, but our current results show that in the presence of cAMP-enhancing agents TGFβ is able to promote osteogenic differentiation. PGE2, which has been shown to raise cAMP levels in hMSCs [53], is known to stimulate osteogenesis upon short-term admission in vivo [54], but it is unclear whether PGE2 exerts its action by preventing TGFβ from inhibiting bone formation. Kim et al. [12] have shown that cAMP-activated protein kinases play a central role in the choice between osteogenic and adipogenic differentiation of hMSCs, but again no correlation was made with the activity of TGFβ. Our present data are summarized in Fig. 7, which show that PPARG, the nuclear transcription factor essential for adipogenic commitment, is inhibited by TGFβ, which consequently suppresses the maturation to adiponectin and Oil Red O-positive fat cells. On its own TGFβ suppresses the activity of RUNX2, the nuclear transcription factor essential for osteogenic commitment by a SMAD3/HDAC5-mediated mechanism [20], resulting in impaired maturation to ALP and matrix mineralization-positive bone cells. However, in the presence of IBMX, which strongly represses HDAC5, TGFβ becomes an activator of RUNX2-mediated osteogenesis. Obviously, the TGFβ-mediated inhibition of adipogenesis is not HDAC5 sensitive.

Using our gene expression microarray analysis we have identified novel inhibitors of adipogenic differentiation. We thereby focused on genes which were differentially expressed on days 1 and 2 following TGFβ treatment. This time frame corresponds with the upregulation of commitment genes for adipogenic differentiation such as PPARG, as shown in Fig. 5. Genes differentially expressed at later time points were particularly involved in fatty acid metabolism (data not shown). Figure 6a shows that under the experimental conditions tested inhibitors of these adipogenic commitment genes did not promote osteogenic differentiation to the same extent as TGFβ. The possibility should therefore be considered that within 24–48 hours the cells have already undergone an irreversible commitment towards either adipogenic or osteogenic differentiation. In that respect it may be interesting to study whether TGFβ added at later time points can still promote cells under adipogenic differentiation conditions to become osteoblasts. Alternatively, the possibility should be considered that under high cAMP conditions TGFβ is able to stimulate specific pathways leading to osteogenic differentiation. Our current results showing that, in the presence of cAMP-enhancing agents, TGFβ is able to prevent the formation of fat cells and promote the formation of bone cells have been obtained on commercially available mesenchymal stem cells from bone marrow of healthy human donors. Given the observation that patients suffering from osteoporosis show an increased number of adipocytes in their bone marrow, it will be interesting to carry out similar experiments with mesenchymal stem cells from such patients. This may indicate if this aberrant pattern of differentiation results from intrinsic changes in their stem cells or from an altered microenvironment in their bone marrow.

## Conclusions

Our data show that in the presence of cAMP-enhancing agents TGFβ stimulates the ability of hMSCs to differentiate into bone cells, while impairing their ability to differentiate into fat cells. Under these conditions TGFβ treatment results in a reduced expression of genes which contribute to adipogenic differentiation, including *PPARG*, *ADAMTS5*, and *AKR1B10*. Since FDA-approved drugs are available for these genes, they are potential targets for treatment of patients suffering from osteoporosis or obesity.

## Abbreviations

ADM, adipogenic differentiation medium; ALP, alkaline phosphatase; BMP, bone morphogenetic protein; DAVID, Database for Annotation, Visualization and Integrated Discovery; DEX, dexamethasone; DMEM, Dulbecco’s modified Eagle’s medium; DMSO, dimethyl sulfoxide; hMSC, human mesenchymal stem cell; IBMX, 3-isobutyl-1-methylxanthine; LIMMA, Linear Models for Microarray Data; MMP, matrix metalloproteinase; ODM, osteogenic differentiation medium; PGE2, prostaglandin E2; PM, proliferation medium; PNPP, *p*-nitrophenyl phosphate; RMA, robust microarray analysis; TGFβ, transforming growth factor beta.

## References

[1] Pino AM, Rosen CJ, Rodriguez JP (2012). In osteoporosis, differentiation of mesenchymal stem cells (MSCs) improves bone marrow adipogenesis. Biol Res.

[2] Justesen J (2001). Adipocyte tissue volume in bone marrow is increased with aging and in patients with osteoporosis. Biogerontology.

[3] Rosen CJ (2009). Marrow fat and the bone microenvironment: developmental, functional, and pathological implications. Crit Rev Eukaryot Gene Expr.

[4] Moerman EJ (2004). Aging activates adipogenic and suppresses osteogenic programs in mesenchymal marrow stroma/stem cells: the role of PPAR-gamma2 transcription factor and TGF-beta/BMP signaling pathways. Aging Cell.

[5] Ito H (2014). Clinical considerations of regenerative medicine in osteoporosis. Curr Osteoporos Rep.

[6] Reid IR (2015). Short-term and long-term effects of osteoporosis therapies. Nat Rev Endocrinol.

[7] Sotoca AM, Weber M, Zoelen EJJ, Daskalaki A (2013). Gene expression regulation underlying osteo-, adipo-, and chondro-genic lineage commitment of human mesenchymal stem cells. Medical Advancements in Aging and Regenerative Technologies: Clinical Tools and Applications.

[8] Muruganandan S, Roman AA, Sinal CJ (2009). Adipocyte differentiation of bone marrow-derived mesenchymal stem cells: cross talk with the osteoblastogenic program. Cell Mol Life Sci.

[9] Bennett CN (2005). Regulation of osteoblastogenesis and bone mass by Wnt10b. Proc Natl Acad Sci U S A.

[10] Roelen BA, Dijke P (2003). Controlling mesenchymal stem cell differentiation by TGFBeta family members. J Orthop Sci.

[11] Rosen ED, MacDougald OA (2006). Adipocyte differentiation from the inside out. Nat Rev Mol Cell Biol.

[12] Kim EK (2012). Human mesenchymal stem cell differentiation to the osteogenic or adipogenic lineage is regulated by AMP-activated protein kinase. J Cell Physiol.

[13] Piek E (2010). Osteo-transcriptomics of human mesenchymal stem cells: accelerated gene expression and osteoblast differentiation induced by vitamin D reveals c-MYC as an enhancer of BMP2-induced osteogenesis. Bone.

[14] Eijssen LM (2013). User-friendly solutions for microarray quality control and pre-processing on ArrayAnalysis.org. Nucleic Acids Res.

[15] Irizarry RA (2003). Exploration, normalization, and summaries of high density oligonucleotide array probe level data. Biostatistics.

[16] Smyth GK (2004). Linear models and empirical bayes methods for assessing differential expression in microarray experiments. Stat Appl Genet Mol Biol.

[17] da Huang W, Sherman BT, Lempicki RA (2009). Systematic and integrative analysis of large gene lists using DAVID bioinformatics resources. Nat Protoc.

[18] da Huang W, Sherman BT, Lempicki RA (2009). Bioinformatics enrichment tools: paths toward the comprehensive functional analysis of large gene lists. Nucleic Acids Res.

[19] Wishart DS (2006). DrugBank: a comprehensive resource for in silico drug discovery and exploration. Nucleic Acids Res.

[20] Kang JS (2005). Repression of Runx2 function by TGF-beta through recruitment of class II histone deacetylases by Smad3. Embo J.

[21] Maroni P (2012). Chemical and genetic blockade of HDACs enhances osteogenic differentiation of human adipose tissue-derived stem cells by oppositely affecting osteogenic and adipogenic transcription factors. Biochem Biophys Res Commun.

[22] Zhou Y, Peng J, Jiang S (2014). Role of histone acetyltransferases and histone deacetylases in adipocyte differentiation and adipogenesis. Eur J Cell Biol.

[23] Bouloumie A (2001). Adipocyte produces matrix metalloproteinases 2 and 9: involvement in adipose differentiation. Diabetes.

[24] Bermeo S, Gunaratnam K, Duque G (2014). Fat and bone interactions. Curr Osteoporos Rep.

[25] Komm BS (2015). The safety and tolerability profile of therapies for the prevention and treatment of osteoporosis in postmenopausal women. Expert Rev Clin Pharmacol.

[26] Devlin MJ, Rosen CJ (2015). The bone-fat interface: basic and clinical implications of marrow adiposity. Lancet Diabetes Endocrinol.

[27] Tuljapurkar SR (2011). Changes in human bone marrow fat content associated with changes in hematopoietic stem cell numbers and cytokine levels with aging. J Anat.

[28] Wrzesinski SH, Wan YY, Flavell RA (2007). Transforming growth factor-beta and the immune response: implications for anticancer therapy. Clin Cancer Res.

[29] Neuzillet C (2015). Targeting the TGFbeta pathway for cancer therapy. Pharmacol Ther..

[30] Santambrogio L (1993). Studies on the mechanisms by which transforming growth factor-beta (TGF-beta) protects against allergic encephalomyelitis. Antagonism between TGF-beta and tumor necrosis factor. J Immunol.

[31] Mori T (1999). Role and interaction of connective tissue growth factor with transforming growth factor-beta in persistent fibrosis: a mouse fibrosis model. J Cell Physiol.

[32] Robinson SP, Rose WC (1992). Transforming growth factor beta 1: lack of in vivo antitumor activity on A549 and Wehi 3BD+ tumors. Anticancer Res.

[33] Blaney Davidson EN, van der Kraan PM, van den Berg WB (2007). TGF-beta and osteoarthritis. Osteoarthritis Cartilage.

[34] Lehrke M, Lazar MA (2005). The many faces of PPARgamma. Cell.

[35] Seals DF, Courtneidge SA (2003). The ADAMs family of metalloproteases: multidomain proteins with multiple functions. Genes Dev.

[36] Durigova M (2011). MMPs are less efficient than ADAMTS5 in cleaving aggrecan core protein. Matrix Biol.

[37] Gorski DJ (2015). Deletion of ADAMTS5 does not affect aggrecan or versican degradation but promotes glucose uptake and proteoglycan synthesis in murine adipose derived stromal cells. Matrix Biol..

[38] Glasson SS (2005). Deletion of active ADAMTS5 prevents cartilage degradation in a murine model of osteoarthritis. Nature.

[39] Rothenberg ML, Nelson AR, Hande KR (1999). New drugs on the horizon: matrix metalloproteinase inhibitors. Stem Cells.

[40] Sparano JA (2004). Randomized phase III trial of marimastat versus placebo in patients with metastatic breast cancer who have responding or stable disease after first-line chemotherapy: Eastern Cooperative Oncology Group trial E2196. J Clin Oncol.

[41] Bourlier V (2005). Protease inhibitor treatments reveal specific involvement of matrix metalloproteinase-9 in human adipocyte differentiation. J Pharmacol Exp Ther.

[42] Khokha R, Murthy A, Weiss A (2013). Metalloproteinases and their natural inhibitors in inflammation and immunity. Nat Rev Immunol.

[43] Mannello F (2006). Natural bio-drugs as matrix metalloproteinase inhibitors: new perspectives on the horizon?. Recent Pat Anticancer Drug Discov.

[44] Penning TM (2015). The aldo-keto reductases (AKRs): overview. Chem Biol Interact..

[45] Zhang W (2014). Knockdown or inhibition of aldo-keto reductase 1B10 inhibits pancreatic carcinoma growth via modulating Kras-E-cadherin pathway. Cancer Lett.

[46] Fukumoto S (2005). Overexpression of the aldo-keto reductase family protein AKR1B10 is highly correlated with smokers’ non-small cell lung carcinomas. Clin Cancer Res.

[47] Liu Z (2012). Epidermal growth factor induces tumour marker AKR1B10 expression through activator protein-1 signalling in hepatocellular carcinoma cells. Biochem J.

[48] Ma J (2008). Aldo-keto reductase family 1 B10 affects fatty acid synthesis by regulating the stability of acetyl-CoA carboxylase-alpha in breast cancer cells. J Biol Chem.

[49] Chalk C, Benstead TJ, Moore F (2007). Aldose reductase inhibitors for the treatment of diabetic polyneuropathy. Cochrane Database Syst Rev.

[50] Liu J, Wen G, Cao D (2009). Aldo-keto reductase family 1 member B1 inhibitors: old drugs with new perspectives. Recent Pat Anticancer Drug Discov.

[51] Matsunaga T (2009). Potent and selective inhibition of the tumor marker AKR1B10 by bisdemethoxycurcumin: probing the active site of the enzyme with molecular modeling and site-directed mutagenesis. Biochem Biophys Res Commun.

[52] Endo S (2010). Selective inhibition of the tumor marker AKR1B10 by antiinflammatory N-phenylanthranilic acids and glycyrrhetic acid. Biol Pharm Bull.

[53] Kleiveland CR, Kassem M, Lea T (2008). Human mesenchymal stem cell proliferation is regulated by PGE2 through differential activation of cAMP-dependent protein kinase isoforms. Exp Cell Res.

[54] Tian XY (2008). Continuous PGE2 leads to net bone loss while intermittent PGE2 leads to net bone gain in lumbar vertebral bodies of adult female rats. Bone.

